# Mycobacterium tuberculosis endocarditis in native valves

**DOI:** 10.11604/pamj.2017.26.194.11515

**Published:** 2017-04-04

**Authors:** Abdelkader Jalil El Hangouche, Latifa Oukerraj

**Affiliations:** 1 Laboratory of Physiology, Faculty of Medicine and Pharmacy of Rabat, Mohamed V University, Rabat, Morocco; 2 Department of Cardiology B, Faculty of Medicine and Pharmacy of Rabat, Mohamed V University, Rabat, Morocco

**Keywords:** Mycobacterium tuberculosis, endocarditis, native valves

## Image in medicine

A 70 year old female presented with a 4 months history of dyspnea, weight loss and fever. The physical examination revealed icterus, multiple lymphadenopathies, hepatomegaly, jugular venous distension and a 2/6 diastolic heart murmur. Laboratory evaluation showed a moderate inflamatory syndrome, cytolysis and negative hemocultures. CT revealed multiple mediastinal and mesenteric lymphadenopathies, segmental thickening of colic wall and ascites. The PPD skin test was positive at 13 mm. The histopathological study of a lymphadenopathy biopsy was compatible with caseum. Quantiferon-TB test was highly positive. HIV serology was negative. A cardiac echocardiography revealed a 28 x 28 mm masse located at the anterior mitral valve (A, B, C, D) fusing to the mitro-aortic junction and along the proximal aortic wall (E, F). The masse was partially drained at left valsalva sinus (G, H). There was a moderate aortic insufficiency (I). The diagnosis of ganglionar tuberculosis with endocadiac and aortic involvement was highly suggested according to clinical, epidemiologic, biologic arguments. The patient was given antituberculosis drugs for 9 months with a spectacular clinical improvement with no regression of the abcess. She is scheduled for surgical treatment.

**Figure 1:**
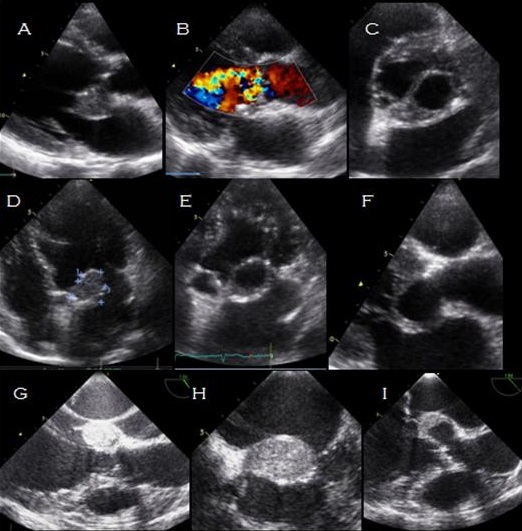
echocardiography images in mycobacterium tuberculosis endocarditis in native valves: A) transthoracic echocardiography, parasternal long-axis view showing a masse located at the anterior mitral valve; B) transthoracic echocardiography, apical 4 chamber view showing the solid part of the mass measuring 23X29mm adherent to the anterior mitral valve; C) transthoracic echocardiography, apical 4 chamber view: showing the empty part of the mass measuring 28X25mm; D) transesophageal echocardiography showing a masse fusing to the mitro-aortic junction; E) transthoracic echocardiography, parasernal short axis view showing a masse fusing in the initial part of the root of the aorta; F) transthoracic echocardiography, parasernal short axis view showing a masse fusing in the the root of the aorta; G) transthoracic echocardiography, parasternal long axis view showing the mass located to the anterior mitral valve and funsig to the mitro aortric junction and along the proximal aortic wall; H) transesophageal echocardiography showing the solid and the empty part of the masse fusing to the mitro-aortic junction, partially drained at left valsalva sinus; I) transthoracic echocardiography, parasternal long axis view moderate aortic insufficiency

